# Clinical Characteristics of an Emergency Department-led Asynchronous Care Platform

**DOI:** 10.5811/westjem.53265

**Published:** 2026-06-28

**Authors:** Shivam Shah, Ikaasa Suri, Helen Gordan, Nicole Schwartz, Rubayet Hossain, Donald Apakama, Rishi Khakhkhar, Taylor Marren, Benjamin S. Abella, Nicholas Gavin, Ethan E. Abbott

**Affiliations:** * Icahn School of Medicine at Mount Sinai, Department of Emergency Medicine, New York, New York; † Icahn School of Medicine at Mount Sinai, New York, New York; ‡ Icahn School of Medicine at Mount Sinai, Department of Population Health Science and Policy, New York, New York; § Icahn School of Medicine at Mount Sinai, Institute for Health Equity Research, New York, New York; ¶ Icahn School of Medicine at Mount Sinai, Charles Bronfman Institute for Personalized Medicine, New York, New York; || Icahn School of Medicine at Mount Sinai, Windreich Department of Artificial Intelligence and Human Health, New York, New York; # Mount Sinai Icahn School of Medicine at Mount Sinai, The Hasso Plattner Institute for Digital Health, New York, New York

## Abstract

**Background:**

Asynchronous care has emerged as a promising approach to improving healthcare accessibility while potentially reducing costs, particularly for low-acuity conditions. Our objective was to describe the use patterns, patient characteristics, and short-term clinical outcomes of an emergency department (ED)–led asynchronous care platform managing low-acuity conditions in a large, academic health system.

**Methods:**

We conducted a retrospective cohort study evaluating the Mount Sinai Health System asynchronous care platform for managing urinary tract infections, conjunctivitis, cold sores, vaginal candidiasis, emergency contraception, and oral contraceptive prescriptions between December 2023–October 2024 during an initial rollout period. Individual charts were reviewed by two trained abstractors and two emergency physicians. The primary outcome was any subsequent ED or clinic visit within seven days. Secondary outcomes included change in diagnosis and medication at relevant follow-up visits. We used descriptive statistics to characterize the cohort and outcomes.

**Results:**

During the study period, there were 19,204 ED encounters for conditions matching asynchronous care chief complaint categories, of which 343 encounters (1.8%) from 277 unique patients were managed via the asynchronous care platform. The median patient age was 32.6 years (interquartile range IQR 28.3–41.2), and 258 (93.1%) were female. Overall chief complaints included cystitis/urinary complaints in 157 (45.8%) encounters, conjunctivitis/eye complaints in 57 (16.6%), and vaginal/vulvar candidiasis in 96 (28.0%). At the encounter level, 18 (5.2%) were up-triaged from the asynchronous care platform to synchronous virtual urgent care or in-person evaluation after initial screening. We observed the primary outcome of a subsequent ED or clinic visit within seven days in 68 patients (24.5%). Five patients (1.5%; 95% CI, 0.5–3.5%) were identified with a change in diagnosis within the seven-day outcome window, with all diagnostic changes occurring within 1–3 days of the index asynchronous care encounter (median one day).

**Conclusion:**

In this single-center study, an asynchronous care platform demonstrated a viable pathway for managing select low-acuity conditions in an urban academic health system. This structured platform, with appropriate triage pathways and low rates of diagnostic discordance, shows promise as a model to expand access to care for select low-acuity complaints.

## INTRODUCTION

The rapid expansion of digital health solutions has reshaped the delivery of healthcare, particularly for ambulatory and emergency care settings. Asynchronous care, a model in which patients and clinicians exchange health information remotely without real-time face-to-face interaction, has emerged as a promising approach to improving accessibility while potentially reducing healthcare costs or emergency department (ED) visits.^1^ This model allows patients to submit structured, symptom-based questionnaires or messages electronically that are rapidly reviewed by a clinician who provides a diagnosis, management plan, or triage decision. Asynchronous care differs from traditional telehealth visits by eliminating the need for synchronous audio or video interactions, offering increased flexibility and efficiency for both patients and clinicians.

The adoption of telemedicine more broadly has been accelerated by the growing burden on EDs and primary care clinics, particularly in the wake of the COVID-19 pandemic.^2^ Prior research has evaluated this surge in synchronous virtual care platforms, demonstrating that live virtual visits can effectively manage a range of low-acuity conditions while reducing ED visits and healthcare expenditures.^3^,^4^ However, within the context of a healthcare system where patients still face barriers to accessing care, asynchronous care offers even greater advantages over synchronous telehealth by eliminating the need for real-time scheduling, prolonged wait times, allowing clinicians to efficiently manage a higher volume of low-acuity cases without disrupting workflow, and affording patients with time constraints or limited broadband connectivity greater flexibility.

Recent evidence has begun to address the safety and efficacy gap for asynchronous care. Reed et al evaluated 24,584 encounters for urinary tract infections (UTI) and conjunctivitis across asynchronous e-visits, telephone visits, and video visits within Kaiser Permanente Northern California.^5^ They found that 69.1% of UTI visits were completed via asynchronous e-visits with comparable seven-day follow-up rates across all modalities (9.0% for e-visits versus 8.8% for telephone and 9.0% for video), and ED visits remained rare (< 1%) with no significant differences between visit types. However, given its novelty, the safety and efficacy of asynchronous care, particularly for common lower acuity complaints, remain less well understood. Key concerns still include diagnostic accuracy, appropriate triage decisions, and potential gaps in follow-up care.

Early evaluations of asynchronous care have shown promising results in managing lower-risk conditions, such as UTIs, conjunctivitis, and dermatologic complaints. 6 Prior research evaluating an asynchronous telemedicine platform for uncomplicated UTIs found that antibiotic prescribing patterns were consistent with guideline-based care and that patient satisfaction was high. 7 In the Kaiser study, the authors found that antibiotic prescribing patterns were consistent with guideline-based care, with 90.7% of UTI e-visit patients receiving antibiotics (reflecting the structured selection of uncomplicated cases) and appropriate variation for conjunctivitis cases. 5 Similarly, an evaluation of asynchronous dermatology consultations demonstrated reduced wait times and high diagnostic concordance with in-person assessments.^8^ These findings suggest that asynchronous care may be a viable and potentially scalable model for specific clinical scenarios. However, concerns persist regarding the potential for misdiagnosis, the adequacy of follow-up care, and the need for escalation to in-person visits.

**Population Health Research Capsule**

What do we already know about this issue?

*Asynchronous care platforms can manage low-acuity conditions like UTIs and conjunctivitis, but safety data from ED-led models remain limited*.

What was the research question?

*Is an ED-led asynchronous care platform safe and feasible for managing low-acuity conditions?*

What was the major finding of the study?

*Of 343 encounters, 1.5% (95% CI, 0.5–3.5%) had diagnostic changes within 7 days; 0.7% had unrelated ED returns*.

How does this improve population health?

*ED-led asynchronous care could safely expand access to care for low-acuity conditions, potentially reducing ED burden and improving healthcare accessibility*.

While prior asynchronous care platform implementations have predominately been led by primary care, to our knowledge this represents a novel approach to asynchronous care delivery with an emergency medicine infrastructure. This ED-led model was selected as the asynchronous care platform is part of a broader digital health portfolio within the Department of Emergency Medicine at Mount Sinai Health System (MSHS), which also includes on-demand virtual urgent care. This existing infrastructure provided a natural foundation for asynchronous care integration, as emergency physicians and physician assistants (PA) already staffed virtual care workflows and are trained in the rapid evaluation of acute, undifferentiated complaints well-suited to the structured, algorithm-driven nature of asynchronous care. This distinction is important as an ED-led asynchronous care model potentially differs from primary care-led models across its integration with emergency triage pathways, ED staffing models, and the potential to divert low-acuity patients from ED visits.

In this descriptive study we sought to examine the use patterns and clinical characteristics of a Department of Emergency Medicine (EM)-led asynchronous care platform during its first year of rollout. The specific objectives were to evaluate feasibility, safety, and clinical outcomes of this ED-led asynchronous care platform by examining rates of up-triage, subsequent visits, and changes in diagnosis, to determine whether asynchronous care managed by emergency physicians and physician assistants can serve as a safe and efficient option for low-acuity conditions in an academic setting.

## METHODS

### Study Design and Setting

We conducted a retrospective cohort study evaluating the asynchronous care platform offered by MSHS, a multisite academic medical center in New York City. This model was developed by the ED to manage visits due to limited access or convenience and was part of a broader digital health initiative by system leadership. This platform is led and staffed by emergency physicians and PAs at MSHS. This model of care is also set up so PAs and emergency physicians are able cross-cover a virtual urgent care system (on-demand video visits for acute, unscheduled care) while also evaluating asynchronous care visits.

In December 2023, MSHS implemented an asynchronous care platform to manage a limited set of chief complaints, including female dysuria, vaginal discharge, and eye redness. In September/October 2024 emergency contraception and birth control prescriptions were added. The platform is marketed to existing MSHS patients through online web banners, portal messages, and hospital marketing. The MSHS serves a diverse patient population through an extensive network of hospitals, ambulatory practices, and specialty institutes located throughout the New York City metropolitan area. In this study we examined electronic health records (EHR) from patients who received an asynchronous online care visit between December 10, 2023–October 21, 2024, which was the initial rollout period.

#### Asynchronous Care Platform Design and User Interface

**Figure 1:**
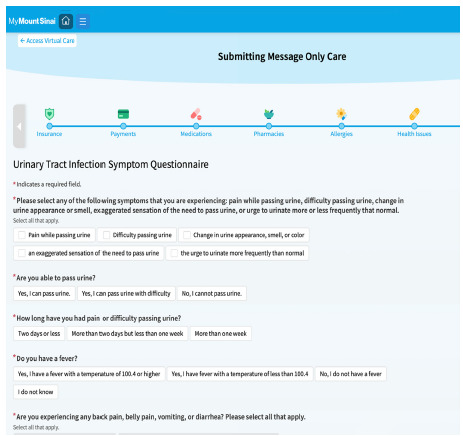
Screening questionnaire for patients with urinary tract infection symptoms in a study describing the use patterns, patient characteristics, and short-term clinical outcomes of an emergency department–led asynchronous care platform managing low-acuity conditions.

To access the platform, adult patients in the MSHS network can opt for “Message Only Care,” which selects the AC option in their online care portal through Epic’s MyChart (Epic Systems Corporation, Verona, WI), which serves as the EHR for the health system. Patients are prompted to complete a condition-specific questionnaire with automated branching logic tailored to their presenting symptoms (Figure 1). The program provides care for a limited set of predefined chief complaints, including UTI, yeast infection, red eye, cold sore, birth control requests, and emergency contraception. Patients who select a chief complaint outside this restricted list are automatically directed to either a video visit or in-person care. Male patients are eligible for treatment of red eye or cold-sore complaints only.

For supported chief complaints, the branching logic incorporates structured screening for predefined high-risk clinical features. Endorsement of any red flag response is automatically highlighted for clinician review and prompts consideration of escalation of care. Specifically, for red eye complaints, high-risk responses include vision loss, double vision, severe eye pain, fever ≥ 101 °F, recent ocular trauma, history of serious eye injury, or autoimmune disease with potential ocular involvement. For UTI evaluations, red flags include fever ≥ 101 °F, flank pain, vomiting, known kidney disease, recent urologic procedure, or inability to void. For suspected vulvovaginal candidiasis, red flag criteria include fever, pelvic or abdominal pain, new sexual partner, possible sexually transmitted infection exposure, or severe or atypical vaginal discharge. Patients who screen as high risk, or for whom asynchronous management is deemed inappropriate upon clinician review, are candidates to be up-triaged to virtual urgent care for real-time video evaluation or directed to in-person care as clinically indicated and at the discretion of the treating clinician.

All electronic submissions are then reviewed by emergency physicians or PAs, who confirm the chief complaint, assess questionnaire responses, and determine the appropriate management plan. Medication prescriptions are selected from a prepopulated, condition-specific list embedded within the asynchronous care platform. Laboratory testing, including urinalysis and urine culture, may be ordered at the discretion of the reviewing clinician. When a visit is up-triaged, it is designated as non-billable and patients are directed to a higher level of care using a standardized communication workflow. At present, the asynchronous care platform operates for patients in New York State and Florida from Monday–Friday 8:30 am – 8:30 pm and Saturday through Sunday 9 am – 5 pm.

### Study Cohort Identification

For this study, adult patients met inclusion if they initiated an asynchronous care visit during the study period through the patient portal. Patients opted into the asynchronous care platform on their own initiative and were not enrolled by ED staff during an ED visit. Patients initiated an asynchronous care visit with a chief complaint of UTI, yeast infection, cold sore, red eye, or request for oral contraceptives or emergency contraceptives prescription. Patients were identified by an MSHS EHR query and filtered for only those who initiated asynchronous care. Within the health system an asynchronous care visit generates a unique patient numerical identifier that is distinct from other types of visits, allowing for capture of this population with high accuracy.

We included all patients who initiated an asynchronous care visit during the rollout period, representing a complete census of the eligible population rather than a sampled subset. Because of this, a formal sample size calculation was not performed, and inclusion was limited to adult patients who initiated an asynchronous care visit with a supported chief complaint during the study period (December 2023–October 2024). We excluded patients who canceled their asynchronous care appointment or whose chief complaint was not supported by the platform. Those who were screened on the asynchronous care platform and subsequently uptriaged to telehealth or referred to the ED or outpatient clinic were included in the initial cohort. As this is a descriptive study without comparative or multivariable analyses, we did not employ methods for handling formal missing data. Where diagnosis or medication data were unavailable, fields were recorded as “unknown.”

### Covariates and Data Abstraction

We collected demographic information (age, sex) and asynchronous visit information (chief complaint, final diagnosis, visit duration, prescribed medications). We identified the following comorbidities using *International Classification of Diseases* ICD-10 codes: asthma; hyperlipidemia; hypertension; diabetes mellitus; and coronary artery disease. Two trained abstractors initially reviewed and screened all asynchronous care visits in the cohort to ascertain whether patients were referred to the ED or follow-up visits within a seven-day period to the ED and clinics for related chief complaints. The index asynchronous care visit date and time was recorded as day 0. For relevant visits within pre-specified interval follow-up visit, we captured final or change in diagnosis, and medications prescribed. To accomplish this, unstructured clinical narratives were reviewed and abstracted from the EHR for all patients in the cohort. This information was confirmed by a third reviewer who was an emergency physician. In equivocal cases, another emergency physician reviewed the data to make the final determination.

The follow-up visits were carefully reviewed to ascertain whether the visit was relevant to the index asynchronous care or it represented a routine/scheduled follow-up or unrelated visit for another chief complaint. For example, if a patient was diagnosed with acute cystitis without hematuria on asynchronous care and had a subsequent clinic visit within seven days with a diagnosis of cystitis or interstitial cystitis, this was classified as a relevant follow up and reviewed for change on diagnosis. In cases where diagnosis and medication information were missing, fields were marked as “unknown.” Patients who followed up outside MSHS were not captured, and we did not include data from health information exchanges in this analysis. Our approach to chart abstraction was informed by recommended methods for medical record review in emergency medicine. We used a standardized abstraction tool with explicitly defined variables, trained abstractors, and physician adjudication of ambiguous cases, consistent with Worster and Bledsoe’s methodological parameters for medical record reviews. 9

### Outcomes

The primary outcome was subsequent ED or clinic visit within seven days related to the index asynchronous care encounter. Secondary outcomes included change in diagnosis and medication at relevant follow-up visits. These outcomes align with prior studies assessing efficacy and safety of asynchronous care. 6,10

### Statistical Analysis

We reported descriptive statistics for continuous variables as means with standard deviations or medians with interquartile ranges. Frequencies with percentages were reported for categorical variables. We performed the analysis using Python version 3 (Python Software Foundation, Wilmington, DE) in MSHS’s secure Minerva supercomputing environment. This study was completed in accordance with the STROBE (Strengthening the Reporting of Observational Studies in Epidemiology) guidelines. The study was approved by the Mount Sinai Institutional Review Board, and a waiver of informed consent was granted for the use of this retrospective dataset (STUDY-24-00779).

## RESULTS

### Overall Cohort

**Figure 2:**
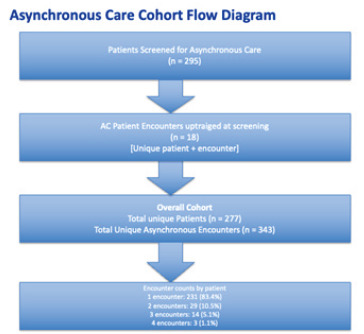
Flow diagram of cohort asynchronous care (AC) encounters in a study of an emergency department–led AC platform in a large urban academic health system, December 10, 2023–October 21, 2024.

We identified 277 unique patients that included a total of 343 encounters to the asynchronous care platform during the study period (Figure 2). At the encounter level, 5.2% (18/343) encounters were up-triaged to virtual urgent care or other care platform after completing initial screening (Table 2). Among the 18 up-triaged encounters, the majority (13/18, 72.2%) were directed to virtual urgent care, with one patient referred to in-person urgent care. The most common chief complaints among up-triaged patients were dysuria (n = 6) and vaginal discharge/vaginitis (n = 4), with up-triage for reasons including presentations outside the asynchronous care scope such as genital herpes simplex, traumatic iritis, and gastrointestinal complaints.

The median patient age was 32.6 years (IQR 28.3–41.2) (Table 1). The sample was predominantly comprised of 258 female patients (93.1%). Of 275 patients, 99.3% reported English as their primary language, and 229 (83.0%) patients reported an active primary care physician in the MSHS. Overall identified comorbidities were low: asthma (13.4%); hyperlipidemia (11.9%); hypertension (10.4%); diabetes mellitus (5.0%); and coronary artery disease (0.5%). Among 343 asynchronous care encounters, the most common chief complaints addressed were cystitis/urinary (45.8%, n = 157), followed by vaginal/vulva/candidiasis (28.0%, n = 96), and conjunctivitis/eye complaints (16.6%, n = 57), while smaller proportions presented for contraception/birth control (2.0%, n = 7). The median visit duration was 12.0 minutes (IQR 6.3–23.5). Overall, no laboratory testing was identified as being sent for patients in the cohort evaluated on the platform. For context, we also ascertained descriptive information (without outcomes) regarding overall in-person ED encounters for the same set of conditions seen on the asynchronous care platform. Concurrently during the study period, there were 19,204 ED encounters with diagnoses matching the asynchronous care chief complaint categories (Table 2). Most of these ED visits were for cystitis/urinary conditions (77.9%, n = 14,956), followed by conjunctivitis/eye complaints (15.7%, n = 3,007) and vaginal/vulva/candidiasis diagnoses (6.0%, n = 1,158), with a small fraction categorized as other diagnoses (0.4%, n = 83).

### Medications Prescribed

A total of 289 prescriptions were dispensed during the study period (Table 4). The most prescribed medications at the encounter level included nitrofurantoin monohydrate representing 30.8% (n = 102) of prescriptions, followed by fluconazole 23.9% (n = 79), and cephalexin at 9.1% (n = 30). Additionally, polymyxin b sulfate 10,000 unit-trimethoprim eye drops accounted for 6.3% (n = 21), and erythromycin ophthalmic comprised 3.9% (n = 13) of prescriptions.

### Asynchronous Care Follow-up Outcomes

Among 277 patients, 68 (24.5%) had a follow-up visit within seven days in the MSHS outpatient system. Two patients (0.7%) presented to the MSHS ED within seven days, both for complaints unrelated to their index AC encounter (adjustment disorder with anxiety and dependent edema, respectively). After comprehensive chart review of all follow-up visits, five patients (1.5%; 95% CI, 0.5–3.5%) were identified with a change in diagnosis, with all changes occurring within 1–3 days of the index encounter (median 1 day) (Table 5). The most common pattern identified was a change in diagnosis between genitourinary complaints: two patients initially treated for presumed acute candidiasis of vulva and vagina were later diagnosed with bacterial vaginosis and prescribed doxycycline and metronidazole; conversely, two patients initially diagnosed with acute cystitis were later diagnosed with vaginal candidiasis and prescribed fluconazole; and one patient initially diagnosed with dysuria was later diagnosed with acute vulvovaginal candidiasis and prescribed fluconazole. No adverse outcomes, hospitalizations, or mortality were noted on chart review among these patients; however, any patients seeking care outside MSHS were not captured.

## DISCUSSION

In this retrospective cohort study, we found that integrating an asynchronous care platform within a large, urban health system provided an effective option for managing common low-acuity conditions and could provide a safe alternative care pathway for select patients. Among the 68 patients who had subsequent follow-up visits within the Mount Sinai Health System, 5 patients (1.5%) underwent a change in diagnosis after their asynchronous visit, and 2 patients were seen in the ED for unrelated complaints. This suggests that this model of care could augment or provide an alternative to traditional face-to-face or synchronous telehealth visits for select low acuity patient complaints if supported by careful clinical workflow design, robust follow-up protocols, and a thoughtful integration into existing health system digital infrastructure.

Our results mirror prior findings on asynchronous care platforms for conditions like uncomplicated urinary tract infections and dermatologic complaints, reinforcing the potential for asynchronous care to expand access and reduce resource utilization in overburdened EDs. A study analyzing electronic visits (eVisit) for minor acute illnesses found that 34% of patients had follow-up within 30 days, reducing to 20% when excluding patients specifically advised to follow-up. Factors significantly associated with follow-up included specific advice from the eVisit clinician and lack of prescription during the eVisit. Four diagnoses (UTI, sinusitis, upper respiratory infection, and conjunctivitis) comprised 87% of all eVisits. Importantly, only 14 patients required ED follow-up, and only one patient was hospitalized within 30 days, with zero deaths reported. 10

A 2023 systematic review specifically examining asynchronous care in general practice found no major safety concerns from 23 studies. This review demonstrated effectiveness in making diagnoses, prescribing medications, replacing other consultation types, providing timely care, and increasing patient convenience. 11 The results of our study also contribute to the growing body of literature evaluating novel digital health interventions and their role in the healthcare landscape. Given the increasing demand for telehealth services and the growing strain on traditional healthcare settings, understanding the utility of asynchronous care models is critical for guiding future implementations and policy decisions. If safe and effective, asynchronous care could offer a scalable solution to expanding access to care while maintaining high standards of patient safety and clinical quality.

Implementation effort and resource requirements are also important considerations for health systems evaluating potential implementation of similar asynchronous care models. The initial build of this asynchronous care platform required substantial clinical leadership effort to develop a framework that supports an excellent user experience for both patients and clinicians. Based on internal estimates, the total platform build cost and implementation cost was approximately $45,000. The estimated cost per individual AC encounter was $21.77, which was calculated based on the average emergency physician assistant salary of $180,000 (plus 24% fringe rate) and the minimum billable encounter time of 11 minutes. The technical effort was moderate and involved building intake, clinical documentation, ordering, billing, coding, and reporting workflows—essentially creating an entirely new visit type within the EHR. However, this work leveraged existing institutional investments in the EHR, patient portal, and telehealth staffing models rather than a standalone build. As a result, we were unable to isolate discrete startup or maintenance costs that would be generalizable to other settings. Once the framework was in place, adding new clinical conditions required relatively minimal incremental effort, focused primarily on building condition-specific intake forms and reaching consensus on “red flag” criteria that would preclude participation. Otherwise, existing workflows were reused.[Table t3-wjem-27-920]

While our study was not designed to directly measure equity outcomes, these findings carry important implications for population health and healthcare equity. Asynchronous care models may reduce barriers to access for patients with a higher burden of unmet health related social needs such as transportation or access to medical care, factors that can disproportionately affect underserved populations. However, we note that the current platform requires digital access, English-language proficiency, and enrollment in the health system’s patient portal, which may inadvertently limit reach to populations with higher digital literacy and connectivity. Future work should prioritize multilingual platform development, targeted outreach to underserved communities, and systematic collection of demographic data to evaluate whether ED-led asynchronous care models narrow existing disparities in healthcare access.

## LIMITATIONS

There are several major limitations to our study. As this study occurred in a single large urban health system, the results may not be generalizable to smaller or rural health systems. Because this study evaluated the initial rollout period of the asynchronous care platform, it may not reflect similar outcomes with a larger sample size. A major limitation is our reliance on internal MSHS follow-up data, which introduced an important source of ascertainment bias, as patients who experienced complications or sought care at outside institutions would not have been captured, likely leading to an underestimation of our outcomes. We also did not validate clinical outcomes beyond chart-documented diagnoses and did not capture patient-reported outcomes such as symptom resolution or satisfaction. Furthermore, this study was not designed to measure the impact of asynchronous care on ED visit volume, crowding, or system-level use, areas to address in future studies.

Additionally, while we reviewed an initial sample of approximately 10% of charts to ensure consistent data capture between abstractors and performed secondary review of all charts with potential misdiagnoses, we did not calculate formal interrater reliability statistics. Given the relatively small number of charts with documented outcomes and the low observed misdiagnosis rate, this represents a potential limitation. We also acknowledge that over 99% of our cohort spoke English as their first language, making our results challenging to generalize to systems with greater proportions of non-English speakers. We also noted a large proportion of unknown category for the race and ethnicity of asynchronous care patients (39.4%), which may limit a comprehensive and granular analysis of use. This also further limits our ability to adequately assess equity in access and outcomes.

Access to the asynchronous care system requires access to the system through a digital device with a connection to the internet. This again would limit access to certain patient populations who may have higher digital and medical literacy and the ability purchase a mobile device or computer. Despite these limitations, the results of this study suggest asynchronous care as a potentially safe and effective tool for health systems and can provide high value care for a limited set of conditions.

## CONCLUSION

In this single-center study, we found effective implementation of an asynchronous care platform within an urban academic health system. A structured platform with appropriate triage pathways demonstrates feasibility as a safe model for managing select low-acuity conditions within an emergency medicine infrastructure, with minimal rates of diagnostic discordance or immediate return visits. Our study demonstrates that integration of an asynchronous care platform within a health system with appropriate safeguards that are carefully designed has the potential to expand access to care for select low-acuity complaints. Further research and randomized clinical trials are needed to confirm our results and understand if a broader set of conditions can be treated in the asynchronous care environment.

## Figures and Tables

**Table 1:** Demographic and clinical characteristics of 277 unique patients and 343 asynchronous care encounters managed on an emergency department–led asynchronous platform at Mount Sinai Health System, December 10, 2023–October 21, 2024.

Characteristic	n	%
Unique patients	277	
Total asynchronous care encounters	343	
Median age, years (IQR)	32.6 (28.3–41.2)	
Sex, n (%)		
Female	258	93.1
Male	19	6.9
Race and ethnicity (self-reported), n (%)		
Non-Hispanic White	103	37.2
Non-Hispanic Black	17	6.1
Hispanic	33	11.9
Asian	2	0.7
Other	13	4.7
Unknown	109	39.4
Age group, years, n (%)		
18–24	17	6.1
25–34	141	50.9
35–44	68	24.5
45–54	21	7.6
55–64	2	0.7
≥65	0	0.0
Active PCP within MSHS, n (%)		
Yes	229	83.0
No	48	17.0
Primary language (self-reported), n (%)		
English	275	99.3
Japanese	1	0.4
Missing	1	0.4
Asynchronous care encounters per patient, n (%)		
1	231	83.4
2	29	10.5
3	14	5.1
4	3	1.1
Chief complaint by encounter, n (%)		
Cystitis/urinary	157	45.8
Vaginal/vulva/candidiasis	96	28.0
Conjunctivitis/eye	57	16.6
Up-triaged	18	5.2
Missing	8	2.3
Contraception/birth control	7	2.0
Comorbidities (patient level), n (%)		
Asthma	27	13.4
Hyperlipidemia	24	11.9
Hypertension	21	10.4
Diabetes mellitus	10	5.0
Coronary artery disease	1	0.5

*IQR* interquartile range*; MSHS*, Mount Sinai Health System*; PCP*, primary care physician.

**Table 2:** Characteristics of encounters up-triaged from asynchronous care (AC) after initial platform screening (n = 18). Patients were up-triaged from the AC platform to synchronous virtual urgent care (vUC), in-person urgent care (UC), or other care settings based on clinical presentation. Up-triage occurred when patients screened positive for high-risk features, presented with complaints outside the AC scope, or were escalated at clinician discretion. Chief complaint and final diagnosis reflect documentation from the AC screening and subsequent care encounter, respectively. Missing data indicate fields not documented in the electronic health record.

Patient	Asynchronous care chief complaint	Final diagnosis	Up-triage destination	Chief complaint category
1	Missing	Acute candidiasis of vulva and vagina	vUC	Vaginal
2	Traumatic iritis	Traumatic iritis	vUC	Eye
3	Acute vaginitis	Acute vaginitis	vUC	Vaginal
4	Bacterial conjunctivitis	Bacterial conjunctivitis	Missing	Eye
5	Dysuria	Dysuria	vUC	Urinary
6	GERD without esophagitis	Polydipsia; UTI, uncomplicated; gross hematuria	Missing	Other
7	Dysuria	Dysuria; ear pain	vUC	Urinary
8	UTI without hematuria	UTI without hematuria	vUC	Urinary
9	Genital herpes simplex	Genital herpes simplex	vUC	Other
10	Acute cystitis without hematuria	Acute cystitis without hematuria	In-person UC	Urinary
11	Dysuria	Dysuria; UTI without hematuria	vUC	Urinary
12	Dysuria	Dysuria	vUC	Urinary
13	Viral URI	Viral URI	Missing	Other
14	Missing	Missing	vUC	Missing
15	Vaginal discharge	Missing	Missing	Vaginal
16	Missing	Missing	vUC	Missing
17	Vaginal discharge	Vaginal discharge	vUC	Vaginal
18	Dysuria	Recurrent UTI	vUC	Urinary

*GERD*, gastroesophageal reflux disease; *UC*, urgent care; *URI*, upper respiratory infection*; UTI*, urinary tract infection; *vUC*, virtual urgent care.

**Table 3 t3-wjem-27-920:** Emergency department encounters and asynchronous care encounters for matched chief complaint categories during the study period (December 10, 2023–October 21, 2024).

Chief complaint category	ED Encounters, n (%)	Asynchronous care encounters, n (%)	Total encounters (ED + Asynchronous care)	Asynchronous care as % of total
Cystitis/urinary	14,956 (77.9%)	157 (45.8%)	15,113	1.0%
Conjunctivitis/eye	3,007 (15.7%)	57 (16.6%)	3,064	1.9%
Vaginal/vulva/candidiasis	1,158 (6.0%)	96 (28.0%)	1,254	7.7%
Contraception/birth control	—	7 (2.0%)	—	—
Uptriaged	—	18 (5.2%)	—	—
Missing	—	8 (2.3%)	—	—
Total	19,204	343 (100%)	19,431*	1.6%*

* Asynchronous care encounters are reported for all 343 unique encounters including up-triaged, missing, and contraception/birth control categories. The asynchronous care as percentage of total column represents the proportion of all encounters (ED + asynchronous care) for each chief complaint category that were managed via the asynchronous care platform.

*ED*, emergency department.

**Table 4:** Medications prescribed during asynchronous care encounters. Total prescriptions = 289.

Medications Prescribed per Encounter		
Medication	n	%
Nitrofurantoin monohydrate 100 mg capsule	102	30.8%
Fluconazole 150 mg tablet	79	23.9%
Cephalexin 500 mg capsule	30	9.1%
Polymyxin b sulfate 10,000 unit-trimethoprim 1 mg/ml eye drops	21	6.3%
Erythromycin 5 mg/gram (0.5 %) eye ointment	13	3.9%
Miconazole nitrate 4 % (200 mg)-2 % vaginal, prefill appl, cream	11	3.3%
Ciprofloxacin 0.3 % eye drops	8	2.4%
Artificial tears 0.3 % eye drops	7	2.1%
Cefpodoxime 100 mg tablet	6	1.8%
Sulfamethoxazole 800 mg-trimethoprim 160 mg tablet	5	1.5%
Olopatadine 0.7 % eye drops	4	1.2%
Valacyclovir 1 gram tablet	3	0.9%
No Rx (No Medications Prescribed)	42	12.8%

Eighteen patients who were up-triaged were not included in this table.

**Table 5:** Patients with change in diagnosis within 7 days. Patients with outpatient follow-up relevant to index asynchronous care encounter with change in diagnosis from index asynchronous care encounter.

Patients with outpatient follow-up and change in diagnosis from AC encounter (n = 5)				
patient	Asynchronous care visit diagnosis	Clinic diagnosis	New medications prescribed	Days to diagnosis change
1	Dysuria	Acute candidiasis of vulva and vagina	fluconazole 150 mg	3
2	Acute candidiasis of vulva and vagina	Bacterial vaginosis	doxycycline hyclate; metronidazole 0.75%	1
3	Acute candidiasis of vulva and vagina	Bacterial vaginosis	doxycycline hyclate; metronidazole 0.75%	1
4	Acute cystitis without hematuria	Acute candidiasis of vulva and vagina	fluconazole 150mg	1
5	Acute cystitis without hematuria	Candida vaginitis	fluconazole 150mg	1
